# Correlations of Plasma Biomarkers and Imaging Characteristics of Cerebral Small Vessel Disease

**DOI:** 10.3390/brainsci14030269

**Published:** 2024-03-12

**Authors:** Qianqian Kong, Xinxin Xie, Ziyue Wang, Yi Zhang, Xirui Zhou, Lingshan Wu, Zhiyuan Yu, Hao Huang, Xiang Luo

**Affiliations:** 1 Department of Neurology, Tongji Hospital, Tongji Medical College, Huazhong University of Science and Technology, Wuhan 430030, China; kqq0203@163.com (Q.K.); xxx0309@163.com (X.X.); ziyue991122@163.com (Z.W.); zhangi0315@163.com (Y.Z.); xg1101735313@126.com (X.Z.); 15893806911@163.com (L.W.); zhiyuan_yu@126.com (Z.Y.); haohuang_tj@hust.edu.cn (H.H.); 2 Hubei Key Laboratory of Neural Injury and Functional Reconstruction, Huazhong University of Science and Technology, Wuhan 430030, China

**Keywords:** cerebral small vessel disease, blood biomarkers, review

## Abstract

Cerebral small vessel disease (CSVD), which is a group of pathological processes affecting cerebral microvessels, leads to functional loss in the elderly population and mostly presents as cognitive impairment and gait decline. CSVD is diagnosed based on brain imaging biomarkers, but blood biomarkers are of great significance for the early diagnosis and progression prediction of CSVD and have become a research focus because of their noninvasiveness and easy accessibility. Notably, many blood biomarkers have been reported to be associated with CSVD in a relatively large population, particularly serum neurofilament light chain (NfL), which has been regarded as a promising biomarker to track the variation trend in WMH and to predict the further status of white matter hyperintensities (WMH) and lacunar infarcts. And neuro-glio-vascular unit structure and blood–brain barrier function have been proposed as underlying mechanisms of CSVD. The article starts from the neuroimaging markers of CSVD, including recent small subcortical infarcts (RSSI), white matter hyperintensities (WMH), lacunes, cerebral microbleeds (CMB), enlarged perivascular spaces (EPVS), cerebral atrophy, and the combined small vessel disease score, and attempts to systematically review and summarize the research progress regarding the blood biomarkers of CSVD that form the changes in the neuro-glio-vascular unit structure and blood–brain barrier function.

## 1. Introduction

Cerebral small vessel disease (CSVD), which is a group of pathological processes affecting cerebral microvessels, leads to functional loss in the elderly population and mostly presents as cognitive impairment and gait decline [1]. CSVD is diagnosed on the basis of neuroimaging markers, including recent small subcortical infarcts (RSSI), white matter hyperintensities (WMH), lacunes, cerebral microbleeds (CMB), enlarged perivascular spaces (EPVS), cerebral atrophy, cortical superficial siderosis, cortical cerebral microinfarct, and the combined small vessel disease score [2]. The emerging neuroimaging findings of CSVD have accelerated the understanding of CSVD pathophysiology and brought opportunities for prevention and treatment ever closer. Notably, some blood markers, such as neurofilament light chain (NfL) and glial fibrillary acidic protein (GFAP), have not been included in the diagnosis of CSVD due to the weak evidence base. However, blood biomarkers are of great significance for the early diagnosis and progression prediction of CSVD and have become a research focus because of their noninvasiveness and easy accessibility.

The neuro-glio-vascular unit structure and blood–brain barrier function have been proposed as underlying mechanisms of CSVD. Pathology studies of CSVD mechanisms describe the concept of the “neuro-glio-vascular unit”, which is formed by neurons, endothelial cells, and glial cells; it contributes to the blood–brain barrier (BBB) function [3] and is important in understanding the pathogenesis of CSVD [1]. Previous MRI studies have demonstrated that BBB dysfunction increases with the increasing WMH burden [4,5] and thus predicts future WMH expansion [6] and incident lacunes [7]. Therefore, changes in the neuro-glio-vascular unit structure and BBB function may play a crucial role in the genesis and development of CSVD.

Based on the aforementioned background, the article starts from the neuroimaging markers of CSVD, including RSSI, WMH, lacunes, CMB, EPVS, cerebral atrophy, and the combined small vessel disease score, and attempts to systematically review and summarize the research progress regarding the blood biomarkers of CSVD that form changes in the neuro-glio-vascular unit structure and blood–brain barrier function.

## 2. Search Strategy

We systematically searched the literature in the PubMed and EMBASE databases up to July 2023, using keywords or MeSH terms (“blood biomarkers”; “Cerebral Small Vessel Diseases”; “Leukoaraiosis”; “microbleed”; “enlarged perivascular space”; “recent small subcortical infarct”; “Atrophy”; “lacune”; and “Cerebral Small Vessel Diseases burden”). There was no limitation on the literature language or publication type. Two authors formulated the inclusion criteria, and all discrepancies were resolved through discussions or by asking a third reviewer. The studies meeting the following criteria were included in the systematic review: (a) they enrolled participants with age-related and vascular risk-factor-related small vessel diseases; (b) they evaluated CSVD neuroimaging markers, including RSSI, WMH, lacunes, CMB, EPVS, cerebral atrophy, and the combined small vessel disease score; and (c) they concerned blood biomarkers, correlated with neuro-glio-vascular unit structure and blood–brain barrier function. The exclusion criteria were: (a) they enrolled participants with multiple sclerosis (MS) and other diseases whose neuroimaging feature was white matter damage that was different from that of WMH in CSVD; (b) they did not discuss arteriolosclerosis-related cerebral small vessel diseases, such as inherited or genetic small vessel diseases, inflammatory and immunologically mediated small vessel diseases, venous collagenosis and post-radiation angiopathy, and non-amyloid microvessel degeneration in Alzheimer’s disease; and (c) they did not involve original research.

Finally, 49 studies were considered eligible for this review, and the important data items were collected (Table 1).

### 2.1. Correlation between Biomarkers and WMH

White matter hyperintensities (WMH), the most studied CSVD neuroimaging marker, represents demyelination, axon loss, and gliosis [1]. Recent studies have shown the significant association between WMH and the dysfunction of endothelial cells and neurons; this is possibly caused by the blocking of oligodendrocyte precursor cell maturation, which impairs myelination and myelin repair [57].

#### 2.1.1. Endothelial Dysfunction-Related Biomarkers

Endothelial failure in CSVD is involved in inflammation, cerebral hypoperfusion, and BBB dysfunction.

Inflammation Some studies have suggested that the neutrophil-to-lymphocyte ratio (NLR) is significantly associated with WMH Fazekas scores and WMH volume [24,47,56], which indicates that NLR may possibly serve as a potential biomarker for WMH. NLR, a marker of the inflammatory response, represents neutrophil aggregation and cytokine release and activation, leading to endothelial dysfunction and white matter damage [58]. In a cohort study, people with high NLR levels had increased odds of atherosclerosis, suggesting that NLR may be a significant feature of atherosclerotic vessels, which may lead to hypoperfusion and worsening WMH volume progression [24]. Additionally, interleukin-6(IL-6) is associated with WMH volume (*p* = 0.01) in a dose-dependent manner [10]. Cystatin C concentration is significantly associated with the severity of WMH (OR = 2.14; *p* = 0.000) [26], and the platelet factor-4 (PF-4) remains associated with the risk of WMH progression (OR = 12.4; *p* = 0.01), even after adjusting for clinically relevant variables (mean arterial pressure, CSVD score, age, sex, and CSVD clinical manifestations) [27]. IL-6, Cystatin C, and PF-4, which are involved in diverse inflammatory processes, are associated with WMH; this has further supported the hypothesis regarding the crucial role of inflammation in WMH mechanisms. Osteopontin (OPN), an extracellular phosphoprotein in response to stress and injury, is upregulated under hypoxic conditions [59], cerebral ischemia [60], and inflammation-associated neurological disease. A recent study has reported the positive correlations between OPN and WMH [41], which are possibly due to the potential neuroprotective effect of OPN [61] and the compensatory response of OPN towards WMH-associated vascular damage and inflammation. Furthermore, a community-based cross-sectional study has shown that the endothelial-related biomarkers, including E-selectin, *p*-selectin, intercellular adhesion molecule 1, vascular cell adhesion molecule 1 (VCAM-1), CD40 ligand, lipoprotein-associated phospholipase A2, chitinase-3-like-1 protein, and total homocysteine (tHCY), are associated with WMH volume (*p* = 0.008) [48], suggesting that endothelial dysfunction may be the bridge between inflammation and WMH.

Many researchers have focused on growth differentiation factor-15 (GDF15), which is believed to be a marker of impaired endothelial function [62]. Higher levels of GDF-15 are significantly associated with the larger WMH volumes [15,19] and poorer cognitive performance [33]. A study has further explored the association between WMH microstructure and GDF15 in a community-based elderly population, concluding that the serum level of MIC-1/GDF15 has a negative association with the average FA value, especially in the corticospinal tract, corpus callosum, superior longitudinal tract, cingulate, and thalamic anterior posterior radiation [14]. The exact mechanism underlying the association of GDF15 with WMH is still a controversial issue. Elevated GDF15, observed in inflammation in damaged tissues [63], may be a marker of a proinflammatory environment that promotes the progression of WMH and the subsequent dementia.

Cerebral hypoperfusion Researchers infer that Aβ may play a role in CSVD pathogenesis due to the important effect of Aβ (amyloid β) on hypertension [64]. A prospective cohort study has demonstrated that Aβ is significantly associated with severe WMH (*p* < 0.05), and it is further reported that plasma Aβ40 is associated with follow-up WMH progression (*p* < 0.05) [50]. The plasma Aβ may enhance endothelium-dependent vasoconstriction and lead to cerebral hypoperfusion, resulting in WMH [65]. Another study has reported that the brain natriuretic peptide (BNP) level is independently associated with WMH (β = 0.722; 95% CI, 0.624–0.819), with an adjustment for clinically relevant variables [31], which may be explained by the possibility that BNP causes a reduction in cerebral blood flow, leading to neurovascular unit dysfunction and WMH.

BBB dysfunction The serum cortisol level has been shown to be associated positively with WMH severity (OR = 1.221, *p* < 0.001) and cognitive impairment (β = −0.154, *p* = 0.001) [50]. An increasing amount of evidence suggests that higher serum cortical levels can downregulate endothelial nitric oxide synthase (eNOS) expression and deactivation and impede nitric oxide (NO) actions, contributing to endothelial and BBB dysfunction [66]. Thus, plasma constituents and the dysfunctional clearance of metabolites leak from tissues and eventually lead to demyelination and gliosis [1]. A study has reported the significant association between the soluble tumor necrosis factor-like weak inducer of apoptosis (sTWEAK) and WMH (*p* < 0.0001) [42]. sTWEAK, a cytokine closely related to endothelial dysfunction, has been proven to change the permeability of the BBB. Researchers infer that sTWEAK contributes to WMH, possibly by inducing the over-expression of proinflammatory cytokines and disrupting the structure and function of the BBB [67]. In addition, the elevated level of antibodies against the NMDA receptor NR2 subunit (NR2ab) is associated with the Fazekas scale of WMH [36]. NR2ab, located in the endothelium of the cerebral arteries [68], potentially leads to excitotoxicity processes that cause damage to the BBB’s integrity [69].

#### 2.1.2. Neurons Dysfunction-Related Biomarkers

Neurofilament light chain (NfL), a neuron-specific and sensitive structural protein [70], is released into CSF and blood following demyelination and axonal damage. Some researchers have regarded plasma NfL as a potential biomarker that can provide valid information about neuroaxonal damage in the central nervous system. Two studies have shown a positive correlation between plasma neurofilament light (NfL) and WMH in non-dementia elderly participants. Notably, a higher baseline plasma NFL concentration is associated with the accelerated progression of WMH; in particular, higher NfL change rates can predict faster WMH progression in the future [34,37]. Similarly, a longitudinal large cohort study has shown that the concentration of log2 (NfL) is significantly associated with the severity and progression of WMH in the follow-up scan [38]. These findings warrant further studies which investigate the potential of plasma NfL as a biomarker to track the variation trend in WMH and to predict the further status of WMH.

### 2.2. Correlation between Biomarkers and EPVS

Perivascular spaces are fluid-filled spaces, where CSF becomes slower and irregular with the increase in arteriolar pulsatility [1]. Researchers have suggested that endothelial dysfunction may impair normal perivascular fluid flushing and the removal of waste by increasing interstitial fluid, which leads to the appearance of enlarged perivascular spaces.

#### Endothelial Dysfunction-Related Biomarkers

Inflammation A study has found that NLR is positively associated with enlarged perivascular space (EPVS) (*p* = 0.017); this indicates that the inflammatory response is involved in EPVS [47]. The possible mechanism is borrowed from multiple sclerosis, which explains why inflammatory cells enter the perivascular space and trigger a series of inflammatory reactions following endothelial cell damage [71].

BBB dysfunction A recent study has described serum cortisol as an independent predictor of moderate to severe EPVS (OR = 1.219, *p* < 0.001) [50]. An elevated serum cortisol level may impair the structure and function of endothelial cells and the BBB, which contributes to the accumulation of amyloid proteins and the failure of protein elimination [72] and consequently leads to EPVS.

### 2.3. Correlation between Biomarkers and Lacunes (of Presumed Vascular Origin)

Studies have shown that the formation of lacunes may be related to the injury of endothelial cells and microglia, representing inflammation, cerebral hypoperfusion, and BBB dysfunction.

#### 2.3.1. Endothelial Dysfunction-Related Biomarkers

Inflammation Some researchers have suggested that local inflammation may contribute to the development and neurological deficits in the so-called ischemic forms of small vessel disease, such as lacunar lesions. And the significant associations between blood inflammatory biomarkers and lacunes, such as OPN [41], homocysteine, and IL-6 [27], provide more evidence of the crucial role of inflammation in lacunes.

Cerebral hypoperfusion Plasma Aβ40 is positively associated with lacunes, and both may predict incident lacunes in the future [30], suggesting that Aβ, a biomarker of endothelium-dependent vasoconstriction and cerebral hypoperfusion, may play a crucial role in the development and progression of lacunes.

BBB dysfunction A study has investigated the possibility that an elevated NMDA receptor NR2 subunit (NR2ab) level is related to the number of lacunes (less than 5) (*p* = 0.039) [36]. The dysfunctional BBB may better explain the potential role of these receptors and the way that they lead to the appearance and development of lacunes [73].

#### 2.3.2. Microglia Dysfunction-Related Biomarkers

In patients with CSVD, microglia are of vital importance in maintaining cerebral vasculature integrity [74]. Tumor necrosis factor-receptor 1 (TNF-R1)-mediated signaling is critical to the regulation of inflammatory responses; it is proposed that it is more reliable than TNF itself because it is detectable for prolonged periods [75]. A recent study has demonstrated that soluble TNF-Receptor 1 (sTNF-R1) is significantly associated with lacunes (OR = 6.91, *p* < 0.001) [53]. This may be explained by the fact that microglia migrate to the ischemic regions, such as lacunes, and by the secretion of chemical attractants, including TNF, which starts the recruitment of inflammatory cells and facilitates the neuroinflammatory reaction. Additionally, the researchers infer that TNF-R1 may have a protective effect on the process of lacunes, in which the upregulation of sTNF-R1 can improve the increases in infarct volume after middle cerebral artery occlusion, according to data from mouse models [76].

### 2.4. Correlation between Biomarkers and CMB

CMB lesions may represent hemosiderin-laden macrophages in perivascular tissue, which is consistent with the vascular leakage of blood cells [2]; therefore, the researchers have focused on the endothelial dysfunction.

#### Endothelial Dysfunction-Related Biomarkers

A study has demonstrated that the mid-regional pro-adrenomedullin (MR-proADM) level is associated with the occurrence of cortical CMBs and the number of CMBs (*p* < 0.01). Furthermore, a higher MR-proADM level is associated with increased odds of having ≥3 CMBs, after adjustment for clinically relevant variables (OR = 2.04, *p* = 0.039) [44]. Firstly, the MR-proADM, a marker of vascular endothelial dysfunction, is associated with CMB, possibly through cerebral vascular vulnerability and cerebral arteriolosclerosis and microinfarctions. In addition, elevated MR pro-ADM levels may be regarded as a secondary response to endothelial injury caused by CSVD, which is accompanied by the pathological deposition of amyloid protein [77].

Another study has suggested that high plasma lipoprotein-associated phospholipase A2 (Lp PLA2) may be a potential and specific biomarker to predict cognitive impairment in CMB patients (ROC = 0.693, *p* < 0.0001), and the number of CMBs significantly mediates the relationship between Lp PLA2 and cognitive decline ((indirect effect = −0.017, *p* = 0.031) [55]. In the central nervous system, Lp-PLA2 induces the loss of pericytes, facilitated by the promotion of oxidative stress and immune responses, which disrupts the blood–brain barrier and allows harmful substances and blood cells to leak into the brain. Therefore, the BBB dysfunction may be involved in the occurrence of CMB, which ultimately leads to the development of cognitive impairment.

In addition, some researchers have focused on the relationship between CMB and the biomarkers of the heart and kidneys. A study enrolling patients with hypertension and CMB has shown that after adjustment for clinical confounding factors, the estimated glomerular filtration rate (eGFR) (OR = 1.95, *p* < 0.05) and the urinary albumin/creatinine ratio (UACR) (OR = 2.25, *p* < 0.01) are independently associated with the prevalence of deep or infratentorial CMBs, while CysC is independently associated with CMBs in the deep or infratentorial (OR = 2.59, *p* < 0.01) and lobar regions (OR = 1.57, *p* < 0.05) [12]. Another study has explored the possibility that patients with higher N-terminal probrain natriuretic peptide (NT proBNP) levels have a greater rate of incident CMBs during a mean follow-up of 2 years (OR = 2.26, *p* < 0.05) [39]. Hypertension partially explains the association between eGFR, UACR, CysC, plasma NT-proBNP, and incident CMBs as hypertension results in damage to the end organs, such as the brain, heart, and kidneys.

### 2.5. Correlation between Biomarkers and RSSI

The recent small subcortical infarcts (RSSI) refer to neuroimaging evidence of recent infarction in the territory of one perforating arteriole. The exact underlying mechanism of RSSI is still a controversial issue and may be associated with endothelial and astrocyte dysfunction and neuron injury [1].

#### 2.5.1. Endothelial Dysfunction-Related Biomarkers

Cerebral hypoperfusion A study has shown that high-sensitivity cardiac troponin T (hs-cTnT) is associated with cortical infarction events (OR = 73.84, *p* < 0.05), independently of demographics and cardiovascular risk factors [39], and possibly results from atrial fibrillation or left ventricular dysfunction, accompanied by cardio-embolism and inflammation. Lacunar infarction is independently associated with BNP levels (β = 0.635, *p* < 0.001), after adjustment for clinical confounding factors [31]; the explanation for this is that the increase in BNP levels may indicate blood stasis, a well-known condition of thrombosis, resulting from the common concurrent drivers of CSVD, such as diabetes mellitus, hypertension, and hyperlipidemia [78]. In addition, elevated cardiac biomarkers levels may represent left ventricular dysfunction, contributing to decreased cardiac output and subsequently affecting cerebral perfusion and RSSI [79].

#### 2.5.2. Astrocytes Dysfunction-Related Biomarkers

Glial fibrillary acidic protein (GFAP), the signature intermediate filament of astrocytes, has been proposed as a potential biomarker in various neurodegenerative disorders including Alzheimer’s disease [80]. Higher baseline serum GFAP (sGFAP) levels have been found in RSSI patients compared to those of controls (187.4 vs. 118.3 pg/mL, *p* < 0.001) [51], with no correlation of the sGFAP levels with the time from symptom onset to baseline blood sampling within 13 days, which indicates that sGFAP is a sensitive marker for acute small ischemic infarcts and is rapidly released into the blood. Blood–brain barrier dysfunction and alterations of the glymphatic system, two promising mechanisms of CSVD, may accelerate GFAP drainage into the blood in RSSI patients, resulting in GFAP being rapidly detected [1].

#### 2.5.3. Neurons Dysfunction-Related Biomarkers

A prospective study has indicated that NfL is positively associated with RSSI at the baseline (73.45 vs. 34.59 pg/mL, *p* < 0.0001); furthermore, it has a significant association with the time from a stroke symptom onset to blood sampling (*p* < 0.0001) [23]. Similarly, another study has found that a higher change rate of NfL can predict the occurrence of lacunar infarcts in the follow-up (OR = 1.99, *p* < 0.001), even after adjusting for demographics, vascular risk factors, cognitive function, and APOE Ɛ4 carrier status [37]. RSSI, representing hypoperfusion of a specific region, may lead to axonal damage of neurons when the neurofilament is released rapidly and detected in the CSF and blood. These findings show that NfL may be a potential and sensitive marker for the assessment of RSSI occurrence and progression and may even benefit the monitoring of treatment responses and prognosis.

### 2.6. Correlation between Biomarkers and Brain Atrophy

Brain atrophy is defined as a lower brain volume, which is not related to a specific macroscopic focal injury such as trauma or infarction [1]. Because of the limited studies, the mechanism of brain atrophy is obscure, but may be partially due to the inflammation and axonal degeneration caused by WMH.

Higher IL-6 levels are reported to be significantly associated with lower gray matter (*p* = 0.001), hippocampal volumes (*p* = 0.012), and increasing CSF volumes (*p* = 0.002), while the associations are similar but weaker for CRP (lower gray matter, *p* = 0.014; hippocampal volumes, *p* = 0.23; and increasing CSF volumes, *p* = 0.067). These findings suggest that inflammation may be involved in brain atrophy in the elderly population [10]. Previous studies have demonstrated that the inflammatory state may participate in the processes of vascular and degenerative diseases and may appear earlier than clinical and neuroimaging manifestations [81].

And there is a significant association between an elevated GDF15 level and decreased total volume of the brain (β = -0.38, *p* < 0.001) and hippocampus (β = -0.003, *p* < 0.05) [33], although its pathophysiology is largely unexplored. GDF15, synthesized by lesioned neurons, plays a crucial role in inflammation in injured tissues [82]. Normalizing GDF15 function may result in the slowing of neuronal loss, possibly by protecting against stress-induced apoptosis [83], suggesting that GDF15 may be a potential therapeutic target to modulate the risk of brain atrophy.

In addition, it has been shown that an elevated OPN level is associated with cerebral atrophy, including central atrophy (OR = 22.2, *p* < 0.05), cortical atrophy (OR = 46.4, *p* < 0.05), and medial temporal lobe atrophy (OR = 49.3, *p* < 0.05), after adjustment for age, gender, education, hypertension, diabetes, and heart disease [41]. Previous studies have paid more attention to the effect of OPN on AD, reporting that the elevation of OPN is observed in the brain and CSF, as well as the plasma of patients with AD [84]. The association between increased OPN levels and brain atrophy in AD suggests that OPN elevation may be a response to neurodegeneration, possibly through the clearance of pathogenic proteins.

### 2.7. Correlation between Biomarkers and Combined Small Vessel Disease Score

Notably, all the neuroimaging markers of CSVD are strictly inter-related, which suggests that the combined small vessel disease score may better capture the general characteristics of CSVD. However, the amount of current research on the combined small vessel disease score is limited. Serum cortisol may be an independent and significant predictor of the total CSVD burden (OR = 1.288, *p* < 0.001), after adjustment for age and sex [50]. And a large cohort study has found that participants with a moderate to severe CSVD burden have higher plasma NfL levels compared to controls (OR = 1.71, *p* = 0.001) at the baseline, and the change rate of NfL has a predictive value for the progression of the CSVD burden in the follow-up (OR = 1.38, *p* = 0.011) [37].

## 3. Discussion

The test of blood biomarkers offers a noninvasive alternative and a method to monitor the severity and track the progression of CSVD neuroimaging markers. Emerging findings point to the role of the blood biomarkers related to neuro-glio-vascular unit structure and blood–brain barrier function in CSVD, which are possibly involved in the processes of inflammation and cerebral hypoperfusion. Earlier studies have focused on endothelial dysfunction-related biomarkers, such as CRP, IL-6, and NLR, which reflect systemic inflammation and are widely reported in various non-central nervous system diseases. Notably, some recent studies have explored and assessed blood biomarkers related to neurons and glial cells, the crucial components of the central nervous system, such as NfL and GFAP. Furthermore, several prospective studies have demonstrated the potential predictive value of NfL and GFAP for the progression of CSVD neuroimaging markers and clinical manifestations, especially cognitive decline. Therefore, more sensitive and specific blood biomarkers associated with the central nervous system are worthy of exploration and verification in populations with CSVD.

In addition, most researchers who explore specific biomarkers based on prior assumptions overlook the fact that CSVD is recognized as being increasingly diverse and that it is probably affected by various and unknown factors. Approaches without previous assumptions, such as omics techniques, might be more appropriate in the exploration of new biomarkers for CSVD, but they are rarely used in the current literature. Joan Jiménez-Balado has used a proteomic approach to find 41 proteins significantly expressed in participants with WMH progression compared to matched controls. Furthermore, neutral ceramidase (ASAH2) is negatively associated with the progression of WMH in the follow-up (*p* = 0.01) [40], which was not reported in the past. ASAH2 prevents the accumulation of ceramides through the sphingolipid metabolism hydrolyzing ceramides pathway [85], which has been shown to be associated with AD neurological pathologies, and consequently affects cognitive function [86]. In addition, Ke-Jin Gao has isolated and identified exosomes from the plasma, finding that miR-320e is an independent predictor of moderate to severe WMH (OR = 0.452, *p* = 0.006) with the potential to be a novel biomarker for CSVD [54]. MiR-320e has been extensively reported in inflammation and oxidative stress injury, and it plays an important role in various ischemic diseases [87]. However, the relationship between miR-320e and CSVD was first revealed, possibly through targeting matrix metalloproteinase-9 (MMP-9) and reducing the extracellular matrix damage, which consequently leads to BBB dysfunction [88]. Accordingly, omics techniques expand the scope of blood biomarkers in CSVD patients and provide clues for further studies that explore the mechanisms of diseases.

The exploration of the blood biomarkers will provide more evidence related to the pathophysiology and mechanism of CSVD. However, the research in humans has identified several manifestations of CSVD, including dysfunctional blood flow and interstitial fluid drainage, especially in the glymphatic system, which may impede the clearance of metabolites from tissues. And researchers have regarded cerebral amyloid angiopathy (CAA) and several monogenic small vessel diseases as protein elimination failure angiopathies [1,89]. Therefore, we should pay more attention to blood biomarkers related to the cerebrospinal fluid (CSF) circulation and glymphatic system, the vital pathway for waste clearance from the neural tissue to maintain normal brain function [90].

Several limitations should be considered when interpreting the results. Firstly, most blood biomarkers can be observed in several disorders, indicating that these biomarkers lack specificity for CSVD diagnosis. Secondly, there is a significant variation in the included studies, with heterogeneity in the criteria of the participants and the potential for publication bias, owing to the observational nature of most studies. Thirdly, the changes in blood biomarkers over time are not assessed and most of studies are cross-sectional, which impedes the further exploration of the longitudinal association between blood biomarkers and CSVD neuroimaging markers. Further prospective studies are required to provide evidence for causality.

## 4. Conclusions

Many blood biomarkers have the potential to be used in the assessment of the severity of CSVD and in the monitoring of the progression of CSVD. More future studies are needed to investigate the longitudinal evaluation of these blood biomarkers for diagnosis and prognosis in CSVD patients. Moreover, studies with a relatively large population-based cohort would be required to confirm the clinical utility of these biomarkers.

## Figures and Tables

**Table 1:** Blood biomarkers with CSVD neuroimaging markers.

Year	Author	Sample	Participants	Blood Biomarkers	Neuroimaging Markers
2009	Giuseppe Licata [8]	46	lacunar stroke	TNF-α, IL-6, IL-1β	RSSI
2011	Elisa Cuadrado-Godia [9]	127	lacunar stroke	vWF, ox-LDL	RSSI
2012	C.L. Satizabal [10]	1841	elderly participants aged 65 to 80 years	IL-6, CRP	WMH, RSSI, brain atrophy
2013	Zachary A. Corbin [11]	405	acute ischemic stroke	MMPs, F2-isoprostane	WMH
2014	Jin-biao Zhang [12]	568	hypertension	eGFR, Cystatin C	CMB
2014	Mitchell S.V. Elkind [13]	1244	lacunar stroke	CRP	RSSI
2015	Jiyang Jiang [14]	327	elderly participants aged 70 to 90 years	MIC-1/GDF15	WMH
2015	Charlotte Andersson [15]	3374	Framingham Offspring	GDF15, ST2	WMH, brain atrophy
2015	Arnab Datta [16]	45	lacunar stroke	proteomic	RSSI
2015	Andrea Vilar-Bergua [17]	972	hypertension	N-glycome Profile	WMH
2015	Stewart J. Wiseman [18]	65	lacunar stroke	inflammation and endothelial activation biomarkers	RSSI
2016	Yuek Ling Chai [19]	324	CIND; AD	GDF15	WMH, RSSI, lacune
2016	Amelia K. Boehme [20]	1244	lacunar stroke	IL-6, amyloid A, TNFR1, CD40L, MCP1	RSSI
2017	Li Yang [21]	56	lacunar stroke	Lipidomic	RSSI
2017	Yanan Zhu [22]	315	CIND; AD t	IL-6, IL-8, TNF-α	WMH, RSSI, lacune
2017	Thomas Gattringer [23]	579	RSSI	NfL	WMH, RSSI
2017	Ki-Woong Nam [24]	2875	people with a health check-up	NLR	WMH
2018	Huimin Fan [25]	389	lacunar stroke	Homocysteine	RSSI
2018	Huang Guoxiang [26]	408	noncritically ill hospitalized patients	Cystatin C	WMH
2018	Jacek Staszewski [27]	123	CSVD	vascular and systemic inflammation biomarkers	WMH, lacune
2018	Daniela Pinter [28]	78	RSSI	NfL	RSSI
2018	Simon R. Cox [29]	593	elderly participants aged 73 to 76 years	S100β	WMH
2018	Esther M.C. van Leijsen [30]	487	CSVD	Aβ	WMH, CMB, lacune
2018	Weimin Wei [31]	346	hypertension	BNP	WMH, CMB, RSSI
2018	Yanan Zhu [32]	310	CIND; AD	HGF	WMH, CMB, RSSI, lacune
2020	Emer R. McGrath [33]	1603	Framingham Offspring	GDF-15, NT-proBNP	WMH, brain atrophy
2020	Yan Sun [34]	1029	CIND	NfL	WMH
2020	Peng Xu [35]	12	lacunar stroke	miR-133, IL-6, IL-8, CRP, TNF-α	RSSI
2021	Larisa A. Dobrynina [36]	70	CSVD	NR2ab	WMH, lacune
2021	Yi Qu [37]	496	CIND	NfL	WMH, CMB, RSSI, CSVD burden
2021	Alison E Fohner [38]	1362	elderly participants aged 65 years or older	NfL, total Tau, GFAP, UCH-L1	WMH
2021	Bibek Gyanwali [39]	434	CIND	NT-proBNP, hs-cTnT, GDF-15	WMH, CMB, RSSI, lacune
2021	Joan Jiménez-Balado [40]	24	hypertension	proteomic	WMH
2021	Yuek Ling Chai [41]	384	CIND; AD	OPN	WMH, brain atrophy
2021	Andres da Silva-Candal [42]	624	hypertension or diabetes w	TWEAK	WMH
2022	Sanne Kuipers [43]	494	Vascular Cognitive Impairment, Carotid Occlusive Disease, heart failure	OLINK cardiovascular III panel	WMH, CMB, RSSI, EPVS
2022	Nagato Kuriyama [44]	214	people with check-up for dementia	MR-proADM	CMB
2022	Stuart J. McCarter [45]	712	elderly participants	Aβ40, Aβ42, t-tau, NfL	CMB
2022	André Huss [46]	42	CSVD	NfL, GFAP	WMH
2022	Yuan Wang [47]	879	CSVD	NLR	WMH, CMB, RSSI, CSVD burden
2022	Ding-Ding Zhang [48]	960	participants aged 35 years or older	systemic, endothelial, and media-related inflammation biomarkers	WMH, CMB, EPVS, lacune
2022	Arnab Datta [49]	62	lacunar stroke	proteomic	RSSI
2022	Qianwen Qiu [50]	158	people with a health check-up	cortisol	WMH, CMB, EPVS, lacune, CSVD burden
2022	Thomas Gattringer [51]	162	RSSI	GFAP	WMH, CMB, RSSI, EPVS, lacune, CSVD burden
2023	Joyce R. Chong [52]	208	CIND; AD	NfL	WMH, CMB, lacune, brain atrophy
2023	Kaung H. T. Salai [53]	206	CIND	TNF-R1	WMH, CMB, RSSI
2023	Ke-Jin Gao [54]	230	CSVD	exosomes	WMH, CSVD burden
2023	Lu Liu [55]	213	CMB	Lp-PLA2	CMB
2023	Shao-Yuan Chuang [56]	720	elderly participants aged 50 years or older	NLR	WMH, CMB, lacune, CSVD burden

RSSI: recent small subcortical infarcts; CSVD: cerebral small vessel disease; CIND: cognitive impairment no dementia; AD: Alzheimer’s disease; MIC-1: macrophage inhibitory cytokine-1; NfL: neurofilament light chain; GFAP: glial fibrillary acidic protein; UCH-L1: ubiquitin carboxy-terminal hydrolase L1; GDF15: growth differentiation factor-15; CRP: C-reactive protein; IL: interleukin; NT-proBNP: N-terminal pro-B-type natriuretic peptide; OPN: osteopontin; TNF: tumor necrosis factor; MMP: matrix metalloproteinase; NLR: neutrophil-to-lymphocyte ratio; NR2ab: Anti-N-methyl-D-aspartate (NMDA) glutamate receptor antibodies; hs-cTnT: high-sensitivity cardiac troponin T; TNF-R1: TNF-Receptor 1; Aβ: Amyloid-β; BNP: brain natriuretic peptide; HGF: hepatocyte growth factor; TWEAK: tumor necrosis factor-like weak inducer of apoptosis; MR-proADM: mid-regional pro-adrenomedullin; Lp-PLA2: lipoprotein-associated phospholipase A2; vWF: von Willebrand factor; ox-LDL: oxidized LDL cholesterol; MCP1: monocyte chemoattractant protein 1.

## Data Availability

Not applicable.
